# Interpersonal distance perception during the normalization of an pandemic situation: Effects of mask‐wearing and vaccination

**DOI:** 10.1002/pchj.719

**Published:** 2023-12-17

**Authors:** Xiaoqing Yu, Chun‐Hsien Chen, Ziqing Xia, Congyi Wang, Wei Xiong

**Affiliations:** ^1^ School of Mechanical and Aerospace Engineering Nanyang Technological University Singapore Singapore; ^2^ School of Design South China University of Technology Guangzhou China

**Keywords:** COVID‐19, face mask, social distance, vaccination, VR technology

## Abstract

This study aimed to evaluate the effect of anti‐pandemic measures, including wearing a face mask and receiving vaccinations, on interpersonal distance (IPD) during the normalization stage of the COVID‐19 pandemic. Virtual reality (VR) technology was used to simulate the experimental environment and a confederate in different conditions. Thirty‐one participants were asked to approach the virtual confederate, who could exhibit three vaccination states and two mask‐wearing conditions, actively and passively in both indoor and outdoor environments. ANOVA results showed that the participants kept a smaller IPD from the confederate wearing a face mask (IPD = 125.6 cm) than from the one without a face mask (IPD = 154.2 cm). The effects of vaccination states were significant, with the largest distance for an unvaccinated confederate (IPD = 182.3 cm) and the smallest distance for the confederate who had received a booster vaccine (IPD = 111.5 cm). Significant effects of environment were also found, with the participants maintaining a larger IPD in an outdoor environment (IPD = 143.4 cm) than in an indoor room (IPD = 136.4 cm). Additionally, the IPD collected when the participants were passively approached (IPD = 149.6 cm) was significantly larger than that obtained when they actively approached the confederate (IPD = 130.3 cm). Moreover, when the participants faced a confederate who had received a booster vaccine and wore a mask, the IPD was not significantly different from that collected before the COVID‐19 pandemic in both the active and passive patterns. These findings help us to better understand the nature of IPD and human behaviors during the normalization stage of the pandemic and provide scientific suggestions for policymakers to develop pandemic‐prevention measures.

## INTRODUCTION

The coronavirus disease 2019 (COVID‐19) pandemic, which was caused by severe acute respiratory syndrome coronavirus‐2 (SARS‐CoV‐2), was first discovered in China in December 2019 and rapidly spread globally. As of 7 February 2022, more than 394.9 million confirmed cases of COVID‐19, including 5.7 million deaths, had been reported (Dong et al., 2020). To protect healthy people and prevent the spread of the virus, the World Health Organization (WHO) recommended a comprehensive package of prevention and control measures, including receiving vaccination, wearing masks, ensuring hand hygiene, and maintaining a physical distance of at least 1 m, etc. (WHO, 2021). An increasing number of researchers are interested in the regulation of interpersonal distance (IPD) after the COVID‐19 outbreak (Calbi et al., 2021; Ebrahimi et al., 2021; Fairlamb & Courtney, 2022; Fini et al., 2021; Mheidly et al., 2020).

In social psychology, the IPD is defined as the emotionally tuned space around individuals where we feel at ease with the proximity of others (not objects) and where we react by increasing distance if we feel threatened or decreasing it if we feel safe in a friendly context (Hall, 1966; Hayduk, 1981; Sommer, 1969). IPD is modulated by individual characteristics such as sex and age (Iachini et al., 2016; Yu et al., 2020), and it is also modulated by situational factors such as temperature perception, background sound, context, etc. (Ruggiero et al., 2019; Tajadura‐Jiménez et al., 2011). People tend to increase IPD in an aggressive context. For example, Vagnoni et al. (2018) suggested that IPD increased more after participants listened to an aggressive conversation than after they listened to a neutral one. Also, a longer distance was found when people saw an angry facial expression than when they saw a happy one (Cartaud, Ott, et al., 2020, Cartaud, Quesque, & Coello, 2020; Ruggiero et al., 2017), which is in accordance with the self‐protection functions ensured by IPD.

Abney (2018) investigated the impact of pre‐pandemic mask‐wearing from physical and social perspectives. The study revealed that masks were associated with being “sick,” leading to a negative attitude during social interactions. Furthermore, in a discussion by Lee and Chen (2021), it was suggested that wearing face masks can generate perceptions of risk, danger, and potential harm. These negative emotions may have increased the IPD even before the COVID‐19 pandemic. However, subsequent studies found evidence suggesting that mask‐wearing could reduce IPD following the outbreak of COVID‐19. Notably, Lisi et al. (2021) observed that participants maintained a significantly smaller distance to individuals wearing face masks. These findings align with the studies conducted by Lee and Chen (2021), Cartaud, Ott, et al. (2020), and Cartaud, Quesque, and Coello (2020), indicating that the pandemic environment and the heightened exposure to wearing masks might influence people's attitude and social behaviors.

While masks and physical distancing are crucial for managing COVID‐19, vaccination is a highly safe and effective method to reduce virus transmission, severe illness, and mortality (Doroftei et al., 2021; Yan et al., 2021). As of 7 February 2022, more than 61.4% of the world's population had received at least one dose of the COVID‐19 vaccine. More than 15 countries have a fully vaccinated rate of over 80% of their populations, while many countries are in the process of obtaining the third booster dose of vaccine, including China and Singapore (Tung et al., 2021). Huang et al. (2021) predicted that physical distancing can be reduced by 36% to 78% as a result of vaccination during social contact in cities with a medium population density. The combination of vaccination and physical distancing could help prevent future COVID‐19 waves. To the best of our knowledge, however, little research has been conducted on the effect of vaccination against COVID‐19 on the psychological IPD that people perceive in social activities. The IPDs that people prefer when they face others with different vaccination states is worth investigating.

Studies showed that the risk of COVID‐19 infection in an outdoor environment was generally lower than that in indoor rooms because of the higher fresh‐air ventilation rate outdoors (Gao et al., 2016; Morawska & Milton, 2020; Rowe et al., 2021). Khosronejad et al. (2020) simulated saliva particle transport during coughing indoors and outdoors, finding that while the maximum spreading length was greater indoors (2.62 m) than outdoors (2.00 m), the spreading speed was higher outdoors owing to mild breeze. Generally, the policies on pandemic prevention and control are more relaxed for outdoor social activities than for indoor ones. For instance, people must engage in indoor exercise activities with masks on but can participate in outdoor exercise with masks off from February 2022 in Singapore (Ministry of Health in Singapore, 2022). Nishihara & Okubo (2015) proposed that there was no significant difference between indoor and outdoor IPDs. However, Welsch et al. (2020) pointed out that spatially limited environments would lead to a temporary tendency to tolerate smaller IPD. Thus, the preferred IPD in indoor and outdoor environments during the COVID‐19 pandemic remains uncertain. Understanding IPD preferences in different settings will be crucial for informing anti‐pandemic policies.

After the COVID‐19 pandemic, Hsieh and Lee (2021) found that the IPD was significantly larger than that before the pandemic. From April 2020, China entered the stage of normalization of the pandemic situation and adopted the strategy of “external defence input and internal defence rebound.” At this stage, the imported cases were basically under control, and the local pandemic situation in China was sporadic, with occasional, small‐scale, clustered pandemics in some areas (Liang et al., 2021, J. Yu et al., 2021). However, very little is known about the regulation of IPD after a series of anti‐pandemic measures such as receiving vaccinations and wearing face masks during the normalization of pandemic prevention and control.

In our previous work, the IPD was collected when participants faced a virtual male confederate in a virtual indoor environment in October 2019, before the outbreak of COVID‐19 (Lee et al., 2021). In the current study, we collected the IPD in the same virtual indoor environment in China in October 2022, when the participants faced the virtual confederate in different anti‐pandemic conditions. In October 2022, China was still in the normalization stage of pandemic prevention and control. The daily new confirmed COVID‐19 cases per million people in the whole of October was less than 1.0 (Mathieu et al., 2020). By 12 October 2022, the ratio of people who had completed the COVID‐19 vaccination protocol had reached 89.35%, while the proportion of people who had received a booster shot in China was 56.65% (Mathieu et al., 2020). The IPDs measured before and after the pandemic will be compared to explore how this psychological distance changes with the pandemic situation and how the anti‐pandemic measures influence the IPD. This will contribute to a deeper understanding of the nature of IPD.

The classical method to measure IPD is based on the judgments of comfort distance. In the passive pattern, the confederate walks towards the participants at a constant speed, and the participants have to stop the approach when they began to feel uncomfortable about the proximity (Adams & Zuckerman, 1991; Han et al., 2023; Hayduk, 1981; Iachini et al., 2014; Yu & Lee, 2020). In the active pattern, participants approach the confederate and stop when discomfort arises (Hecht et al., 2019; Hsieh & Lee, 2021). VR technology has gained popularity in research involving psychology (Iachini et al., 2016; Xiong et al., 2022; Yang et al., 2023), offering a promising tool for reducing infection risk during pandemics. Moreover, VR provides an immersive experience and allows precise control over experimental settings, including virtual confederates' characteristics and behaviours (e.g., walking speed, eye gaze, and mask‐wearing).

As mentioned above, pandemic‐prevention measures are comprehensive (including wearing masks, maintaining physical distance, receiving vaccinations, etc.), and there is a lack of studies that comprehensively consider the influence of various anti‐pandemic measures on IPD in both indoor and outdoor environments. Therefore, this study aims to investigate the influence of anti‐pandemic measures on IPD in the normalization stage of the COVID‐19 pandemic using the VR simulation method. More specifically, the purposes of this study are (1) to evaluate the impact of mask‐wearing and vaccination on IPD in people's social interactions during the pandemic, (2) to examine the IPD that people chose in indoor and outdoor environments during the pandemic, and (3) to compare the IPD collected before and after the COVID‐19 pandemic. We propose four major hypotheses. First, anti‐pandemic measures, including mask‐wearing and vaccination, reduce IPD; second, participants choose a shorter IPD in an indoor room than in an outdoor square; third, participants prefer a larger IPD when passively approached than when actively approached; fourth, anti‐pandemic measures help restore the IPD under normalized pandemic conditions to pre‐pandemic levels. This study will contribute to a deeper understanding of IPD in the COVID‐19 environment and generate important insights into psychological responses in people's social activities under different anti‐pandemic measures.

## MATERIALS AND METHODS

### Participants

Before the data collection, G*Power was used to conduct a prior power analysis (Faul et al., 2007). The result showed that at least 18 participants were required to achieve a power of 95% with an effect size of 0.20. Thirty‐one participants (16 males) aged 19–27 years old (*M* = 21.4, *SD* = 1.9) were recruited for this study. All the participants were right‐handed, and they all had normal or corrected‐to‐normal vision. The average height and weight for the male participants were 176.2 ± 6.7 cm and 62.1 ± 7.9 kg, respectively. The height and weight for the female participants were 164.1 ± 4.3 cm and 54.6 ± 7.4 kg, respectively. As of the beginning of the experiment, all the participants had received a booster vaccine. Additionally, none of the participants had ever been infected with COVID‐19, and none of their relatives or loved ones had experienced a severe case of COVID‐19 or had died. No cognitive impairment or other illnesses that might affect spatial perception were found among the participants (Silton et al., 2011). According to the self‐reporting, all the participants had no prior knowledge about the purpose of the experiment. Each participant signed an informed consent form before the data collection.

### Virtual environmental settings

**FIGURE 1:**
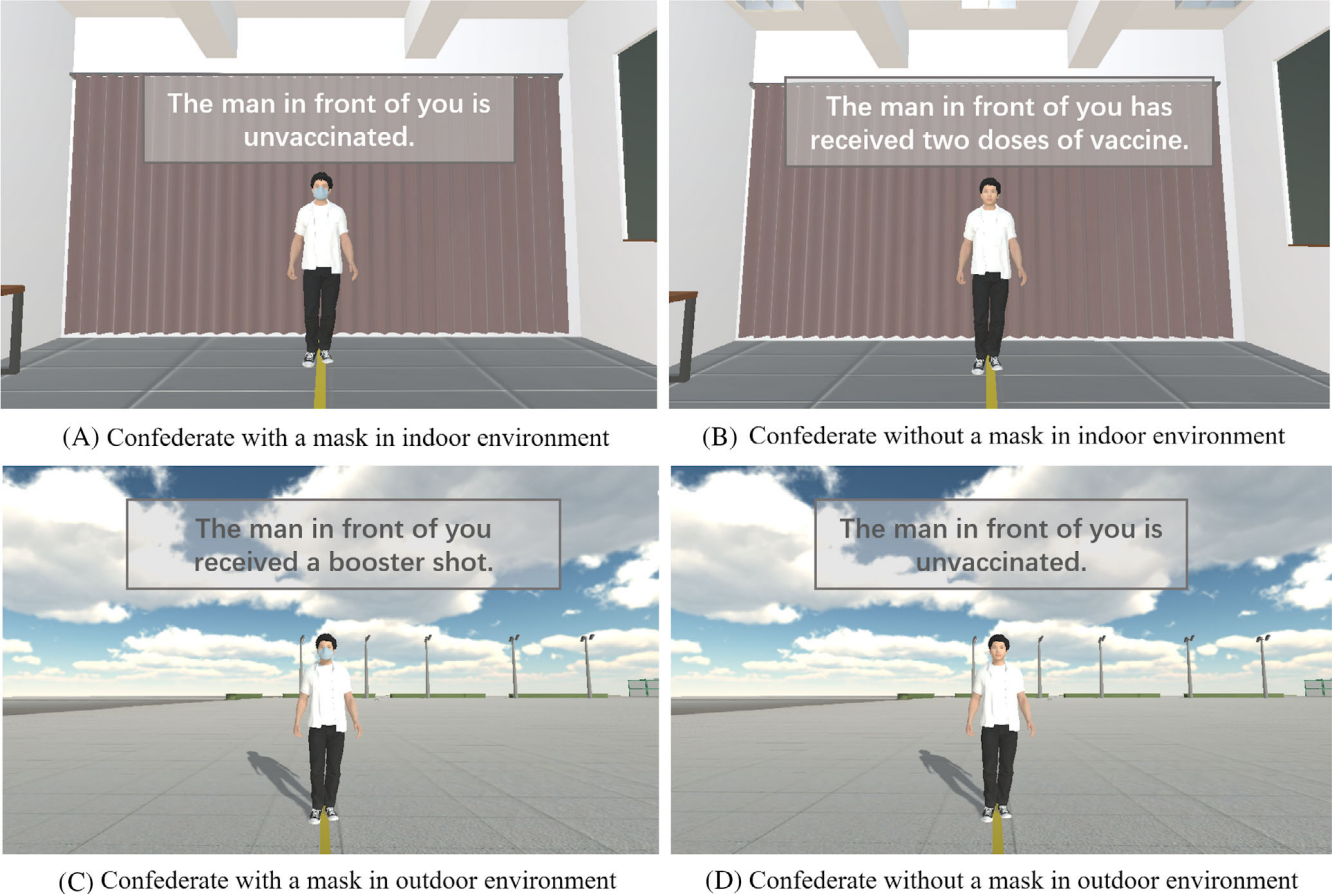
Examples of experimental settings in this study.

Two virtual environments, an indoor room and an outdoor square, were built in the Unity 3D game engine (Unity Technology, San Francisco, CA, USA), which can be seen in Figure 1. A virtual male confederate in a white t‐shirt and black jeans without any accessories was built by 3Ds Max (Autodesk, San Francisco, CA, USA). The virtual confederate was 175 cm tall, had a medium stature, and maintained a neutral facial expression. The VR equipment used in this experiment was Oculus Quest 2 (Meta Platforms, Menlo Park, CA, USA), which included a head‐mounted display (two AMOLED panels) and two hand controllers. The resolution of each AMOLED panel was 1832 × 1920 (3664 × 3840 for combined panels), and the refresh rate of the headset was 90 Hz. The experiment was carried out in a cuboid room (8 m × 6 m × 4 m) without sundries. When the participants wore the head‐mounted display, they felt immersed in a virtual environment (either an indoor room or an outdoor square). Four meters in front of them stood a virtual male confederate with or without a face mask. The mask used in this experiment was a common blue surgical mask without any decoration, which was the type most commonly used during the COVID‐19 pandemic. There were three states of vaccination for this confederate: (1) unvaccinated; (2) received two doses of vaccine; and (3) received a booster shot (see Figure 1). A yellow line was marked on the ground to guide the approaching movement. The participants could walk freely in the virtual environment, and they could trigger or stop the virtual confederate's movement by pressing the button on the hand controller.

### Procedure

**FIGURE 2:**
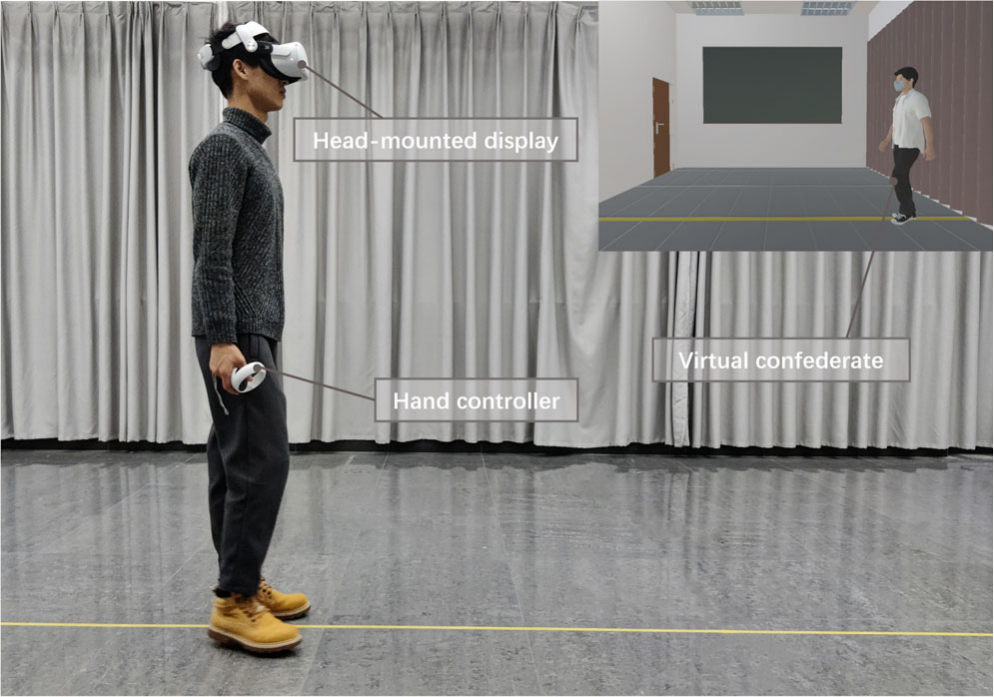
The experimental scene and the virtual experiment room.

When the participants arrived at the experimental laboratory, each received written instructions and signed the informed consent form. Basic personal information was collected by self‐reporting. The experimenter then orally introduced the entire experimental procedure to the participants. Before the data collection, the experimenter showed the participants how to use the VR equipment and helped them to wear the head‐mounted display correctly. When the participants wore the VR headset, the real world was invisible (as shown in Figure 2). To familiarize the participants with the VR environment, they were encouraged to freely walk in the virtual environment and observe the virtual confederate for 5 min. In the process of immersive exploration, the participants were asked to describe their feelings about the presence of the virtual environment and virtual confederate. All the participants reported that they felt like they were in a real square/room and facing a real person.

The starting point, where the participants were initially located, was 4 m away from the virtual confederate (Yu et al., 2020). Hand controllers were provided for the participants to control the virtual confederate's movement. Two practice trials were conducted before the formal experiment to familiarize the participants with the distance judgment procedure. To avoid learning effects, the virtual confederate used during the training phase was different from the one used in the formal experiment. In the formal experiment, the virtual confederate was designed in 3 (unvaccinated vs. two doses vs. a booster shot) × 2 (mask on vs. mask off) conditions. Each participant was required to judge the IPD when they actively and passively approached the confederate in each of six conditions. The above judgment procedure was repeated in both indoor and outdoor environments. The virtual confederate's vaccination state was provided by placing a label above the confederate's head for 10 seconds at the beginning of each trial. For the active approach pattern, the participants were asked to walk towards the virtual confederate and stop when they began to feel uncomfortable. In the passive pattern, the confederate walked towards the participant at a constant speed of 0.5 m/s. When the participants felt uncomfortable, they pressed the button on the hand controller to stop the confederate's approach. After the confederate stopped, the experimenter asked the participants whether they were happy with confederate's position. If they were not satisfied with this position, they had an opportunity to slightly adjust it. After the participants confirmed the stop position, the horizontal distance between the virtual confederate and the participant was automatedly calculated and recorded by the computer program (Lee et al., 2021). The experimenter have to guarantee that the procedure was the same for all participants after each single trial. Each trial was repeated twice to avoid accidental error, and the average value was calculated for further analysis. A 10‐minute break was provided for each participant between the experiments in the indoor and outdoor environments to avoid perception fatigue. During the whole procedure, the experimenter guided and helped the participants when necessary. Each participant conducted 48 trials (3 vaccine states × 2 mask‐wearing × 2 environments × 2 patterns × 2 repetitions) in total. The order of the trials was randomly assigned in the VR program. For each participant's trial, the VR program automatedly generated a confederate under one of 24 conditions, and the probabilities of the appearances of all the conditions were set to be equal.

### Statistical analysis

The average value of the repeated measurements was considered as the IPD for each trial, measured in centimeters. This study conducted a full‐factorial experiment with four within‐subject factors. The independent variables were mask‐wearing (with or without), vaccination states (unvaccinated, received two doses, or added a booster shot), environment (indoor or outdoor), and approach patterns (active or passive). The data were then analysed using SPSS 26.0 (SPSS Inc., Chicago, IL, USA) with a significance level of 0.05. A four‐way repeated‐measures analysis of variance (ANOVA) was conducted to evaluate the effects of the independent variables on the IPD, and the Bonferroni pairwise comparison test was used to compare the post hoc significant effects. Furthermore, the independent *t‐*test was used to compare the distance collected in this study and the data collected before the COVID‐19 pandemic in our previous study (Lee et al., 2021), so that we could evaluate the regulation of IPD with the change of pandemic status and implementation of pandemic‐prevention measures.

## RESULTS

**FIGURE 3:**
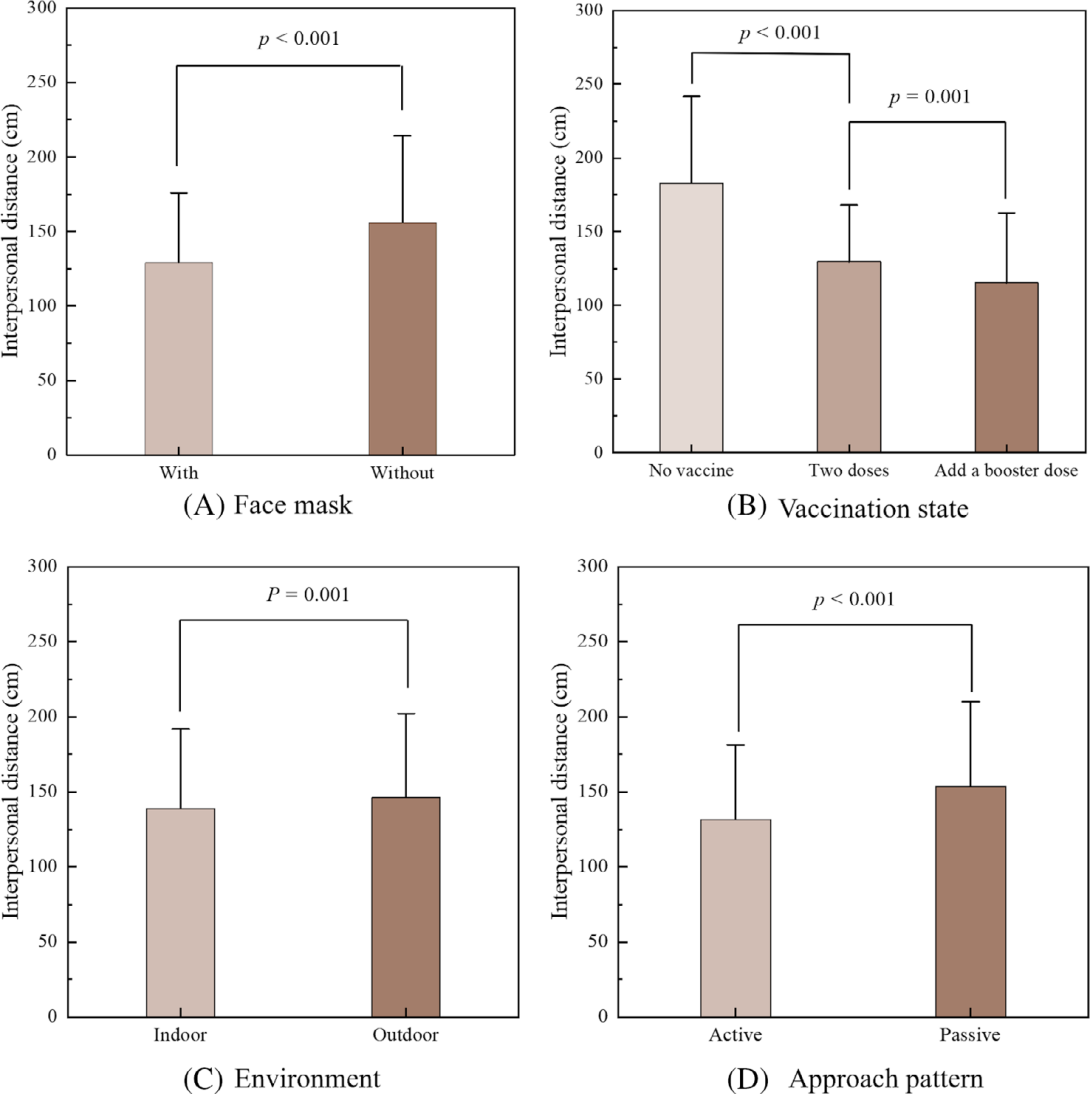
The average interpersonal distances of participants for the four main effects.

Before conducting the ANOVA, the IPDs in 24 conditions were checked for normality of distribution. The results of the Shapiro–Wilk test indicated that the IPDs in each condition were all normally distributed (all *p*s > .05). The four‐way repeated‐measures ANOVA results for IPD are summarized in Table 1. The results show a significant main effect of the face mask [*F* (1,25)= 69.337, *p* < .001, η^2^ = 0.735] on IPD. The Bonferroni post‐hoc test showed that the participants kept a significantly larger IPD from the virtual confederate without a face mask (*M* = 154.2 cm, *SD* = 58.2 cm) than from the one with a face mask (*M* = 125.6 cm, *SD* = 46.8 cm) [*t* (25) = 8.326, *p* < .001, Figure 3A]. A significant effect of vaccination state on IPD was found [*F* (2,50)= 77.351, *p* < .001, η^2^ = 0.710]. According to the Bonferroni pairwise comparison, the participants maintained a significantly larger IPD when they faced the confederate who was unvaccinated than when they faced the one who had received two doses, with a difference of 56.46 ± 7.29 cm [*t* (24) = 7.741, *p* < .001, Figure 3B]. The difference between the IPD when two vaccinations had been received and that when a booster dose had been added was 14.32 ± 4.36 cm [*t* (24) = 3.288, *p* < .01]. Additionally, the effect of the environment on the perception of IPD was significant [*F* (1,25)= 12.912, *p* = .001, η^2^ = 0.341]. The IPD obtained in the virtual indoor room (*M* = 136.4 cm, *SD* = 52.9 cm) was significantly smaller than that obtained in the outdoor environment (*M* = 143.4 cm, *SD* = 55.9 cm) [*t* (25) = 3.593, *p* = .001], as illustrated in Figure 3(C). Furthermore, a significant effect of the approaching pattern [*F* (1,25) = 27.115, *p* < .001, η^2^ = 0.520] showed that the participants chose a larger distance when they were passively approached than when they actively approached the confederate, with a difference of 19.29 ± 3.71 cm [*t* (25) = 5.207, *p* < .001, Figure 3D].

**TABLE 1:** The results of the four‐way analysis of variance on interpersonal distance.

Effects	*df*	*F*‐value	*p*‐value	η^2^
Face mask	1	69.337	<.001	0.735
Vaccination	2	61.280	<.001	0.710
Environment	1	12.912	.001	0.341
Approach	1	27.115	<.001	0.520
Vaccination × Face mask	2	7.145	.01	0.222
Vaccination × Environment	2	0.336	.716	‐
Vaccination × Approach	2	0.753	.476	‐
Face mask × Environment	1	0.186	.670	‐
Face mask × Approach	1	1.906	.180	‐
Environment × Approach	1	0.273	.606	‐
Vaccination × Face mask × Environment	2	0.046	.955	‐
Vaccination × Face mask × Approach	2	1.264	.291	‐
Vaccination × Environment × Approach	2	0.388	.680	‐
Face mask × Environment × Approach	1	0.435	.515	‐
Vaccination × Face mask × Environment × Approach	2	1.362	.266	‐

**FIGURE 4:**
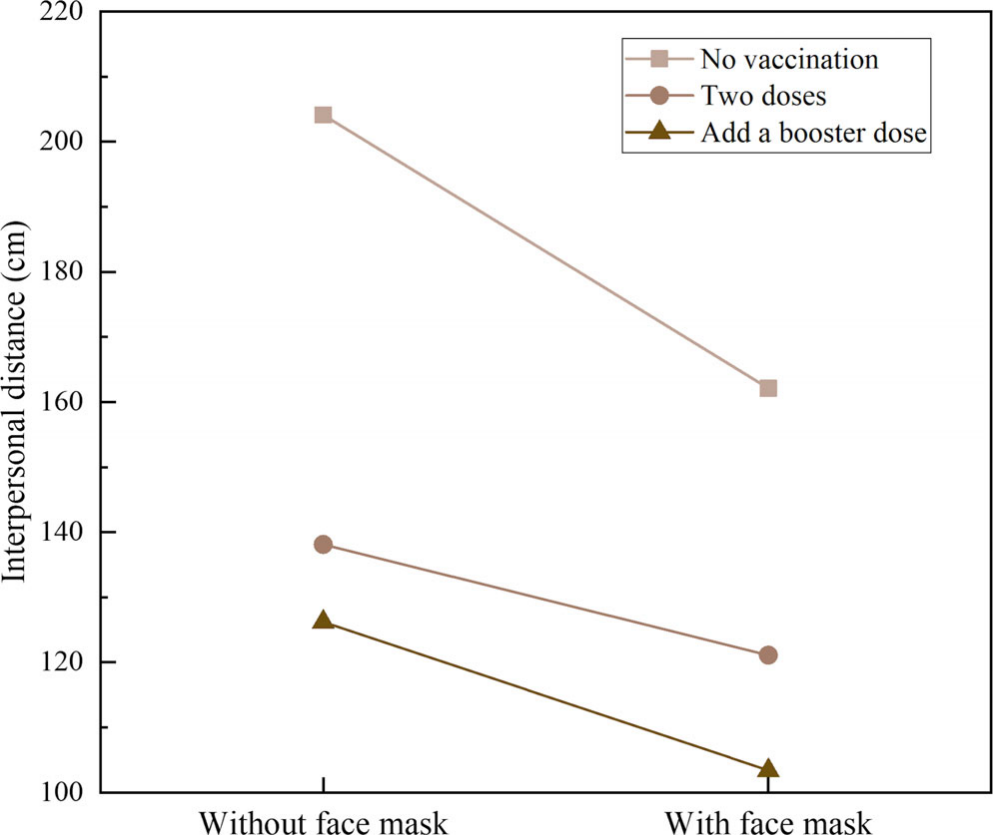
Interaction effects between vaccination and face mask on interpersonal distance.

Moreover, the interaction effect between vaccination states and the face mask was significant [*F* (2,50) = 7.145, *p* < .01, η^2^ = 0.222] (see Figure 4). According to the results for the simple effect of face mask, when facing an unvaccinated confederate, the participants kept a smaller distance from the confederate wearing a face mask than from the one without a face mask [*t* (25) = 6.444, *p* < .001]. Similarly, in the condition of the confederate having received two doses of vaccine, the IPD was smaller when the confederate wore a face mask than when they did not [*t* (25) = 3.833, *p* = .001]. Additionally, when the confederate had received a booster dose, there was a significantly smaller IPD in the mask‐wearing condition than in the no‐mask condition [*t* (25) = 5.830, *p* < .001]. For the simple effect of vaccination, when the participants faced the confederate wearing a face mask, the IPD that was kept from the unvaccinated confederate was significantly larger than the one that was kept from the confederate who had received two doses of vaccine, with a difference of 44.02 ± 6.94 cm [*t* (25) = 6.343, *p* < .001]. The IPD was larger when the confederate had received two doses of vaccine than when a booster shot had been added, and the difference was 15.75 ± 3.85 cm [*t* (25) = 4.091, *p* = .001]. When the participants faced the confederate without a face mask, there was no significant difference between the states of receiving two doses of vaccination and adding a booster dose (*p* > .05). However, the participants kept a significantly larger IPD from the unvaccinated confederate than from the confederate with two doses of vaccination in the no‐mask condition [*t* (25) = 7.198, *p* < .001], and the difference was quite large (68.89 ± 9.57 cm). Overall, when the confederate was unvaccinated and did not wear a face mask, the participants chose the largest IPD of all the conditions (*p* < .001).

In our previous study that was conducted in October 2019 (before the first COVID‐19 outbreak in December 2019), 40 participants were recruited to conduct an experiment of measuring the IPD in the same virtual room as in this study. As there was no pandemic at that time, the participants and the virtual confederate did not wear face masks and were all unvaccinated. The participants were required to judge the IPD when they actively and passively approached the virtual character. The collected IPDs in the active and passive patterns were 89.0 ± 18.4 cm and 107.2 ± 18.1 cm, respectively. The IPD collected in the indoor room in this study was chosen to be compared with that collected before the pandemic. A total of 12 sets of data were selected (2 face masks × 3 vaccination states × 2 approach patterns).

**FIGURE 5:**
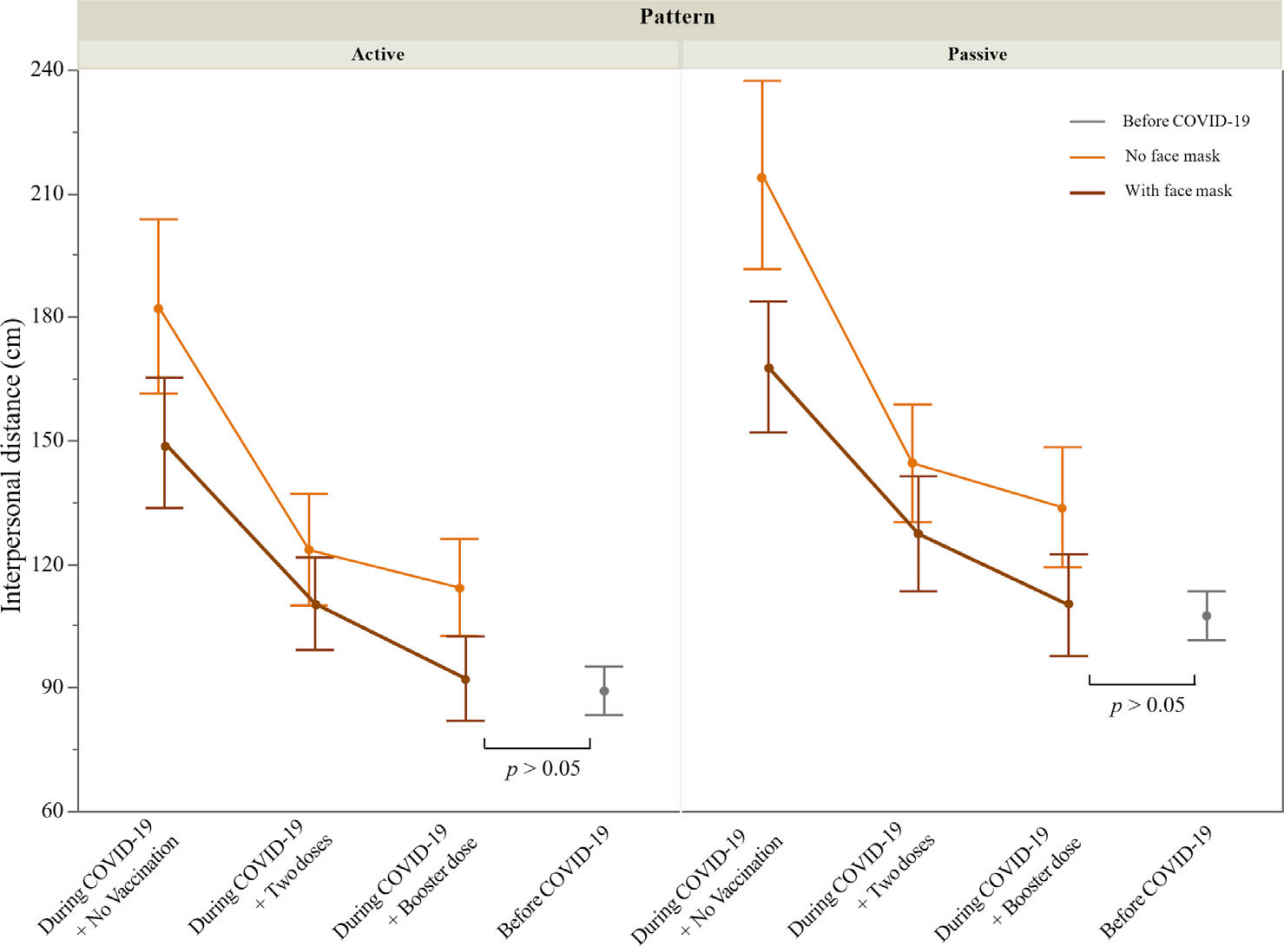
Comparison *t*‐test of interpersonal distance before and during the COVID‐19 pandemic.

Before conducting the independent *t*‐test, the IPDs collected before and after the pandemic were each subjected to a Shapiro–Wilk test to assess the normality of their distribution. The results of the tests were all nonsignificant (*p* > .05), suggesting that the data follow a normal distribution. The results of the independent *t*‐test on the IPD data collected before and during the pandemic are shown in Table 2 (Lee et al., 2021). As can be seen in Figure 5, in the active pattern, participants maintained a significantly larger IPD during the COVID‐19 pandemic compared with before the pandemic, in all conditions (all *p*s < .01), except when facing a confederate wearing a face mask and having received a booster dose. It is noteworthy that there was no significant difference between the IPDs collected before the COVID‐19 pandemic (*M* = 89.0 cm, *SD* = 18.4 cm) and when participants faced a virtual confederate wearing a face mask and having received a third booster dose (*M* = 92.0 cm, *SD* = 27.8 cm) (*p* > .05). Similarly, the IPD when participants were approached by the confederate wearing a mask and having received a booster shot (*M* = 109.9 cm, *SD* = 33.0 cm) was not significantly different from the IPD before the pandemic (*M* = 107.2 cm, *SD* = 18.1 cm) (*p* > .05). However, significantly larger IPDs were observed in the other five conditions of anti‐pandemic measures during the COVID‐19 pandemic compared with the distances collected before the pandemic (all *p*s < .05).

**TABLE 2:** The results of the independent *t* test on interpersonal distance (IPD; cm) before and during the COVID‐19 pandemic.

Pattern	Before COVID‐19	During COVID‐19			Independent *t* test		
	IPD	Face mask	Vaccination	IPD	*t*	*df*	*p*‐value
Active pattern	89.0 (18.4)	Without face mask	No vaccination	182.4 (57.8)	8.655	68	.001
			Two doses	123.3 (36.9)	4.718	68	.001
			Booster dose	114.1 (32.6)	3.872	69	.001
	89.0 (18.4)	With face mask	No vaccination	149.3 (43.2)	7.261	68	.001
			Two doses	110.2 (30.7)	3.381	68	.01
			Booster dose	92.0 (27.8)	0.507	68	.614
Passive pattern	107.2 (18.1)	Without face mask	No vaccination	214.3 (62.2)	9.269	68	.001
			Two doses	144.3 (38.8)	4.910	68	.001
			Booster dose	133.6 (40.3)	3.429	69	.01
	107.2 (18.1)	With face mask	No vaccination	167.6 (43.4)	7.261	68	.001
			Two doses	127.1 (38.3)	2.667	68	.05
			Booster dose	109.9 (33.0)	0.397	67	.693

*Note*: The IPD is shown as the mean (standard deviation).

## DISCUSSION

This study found that the participants preferred a smaller IPD when they approached a confederate wearing a face mask, which is consistent with the results of studies conducted in the early stage of the COVID‐19 pandemic (Cartaud, Ott, et al., 2020, Cartaud, Quesque, & Coello, 2020; Lee & Chen, 2021; Mheidly et al., 2020). The participants reported that they experienced a strong feeling of unsafety and invasion when they faced a confederate without a face mask. However, this result is inconsistent with people's negative attitude towards masks before the pandemic. This inconsistency can be explained by considering that the pandemic environment and the heightened exposure to anti‐pandemic measures might change people's attitude towards mask wearing. In addition, this study was completed in China. Zhao and Knobel's (2021) study proposed that Chinese participants demonstrated a greater pro‐masking tendency than Europeans. Similarly, Wang et al. (2020) found that the proportion of Chinese participants wearing masks (96.8%) was much higher than that of Polish participants wearing masks (35.0%), which showed a higher acceptancy of wearing masks in China. Therefore, wearing a face mask during a pandemic will bring a sense of security and a low risk of infection, leading to a smaller IPD for Chinese participants.

The role of a vaccine in controlling the COVID‐19 pandemic depends not only on the safety and efficacy of the vaccine but also on public acceptance of vaccination (Sallam, 2021). Although vaccine hesitancy, which is described as delayed acceptance or rejection when a vaccine is available, exists in many areas and countries, Sallam (2021) summarized that the acceptance of vaccines was generally greater than 70% in most studies. The current study suggested that the participants maintained the largest IPD when they faced an unvaccinated confederate, while the closest distance was found when they faced a confederate who had received a booster shot. This result is consistent with the self‐defence nature of IPD (Huang & Izumi, 2021; Ruggiero et al., 2017). When they faced an unvaccinated confederate, the participants were concerned that the confederate was unsafe and at risk of carrying the virus, leading to quite a large social distance. It can be assumed that people exhibited high trust in and acceptance of the effectiveness of the vaccine. The significant interaction effects between vaccination states and face masks showed that wearing a face mask would reduce the IPD and that the IPD would simultaneously be affected by vaccination states. The participants kept the largest IPD in all the cases of mask‐wearing and vaccination states when the confederate was unmasked and unvaccinated, indicating that the participants felt the least secure in the absence of protection measures. The findings demonstrate that anti‐pandemic measures, including wearing face masks and the popularity of vaccination, could jointly regulate people's social behaviors and activities.

This study also evaluated the participants' IPD in both indoor and outdoor environments during the COVID‐19 pandemic. The results showed that the participants chose a smaller IPD in the indoor room than in the outdoor square. This phenomenon is consistent with findings by Welsch et al. (2020), who pointed out that constrained spaces typically encouraged people to temporarily tolerate smaller IPD, and thus participants tended to choose a smaller IPD in a restricted, indoor room. However, studies have shown that the risk of infection in indoor rooms is higher than that in outdoor spaces because the air ventilation rate in indoor rooms is lower (Gao et al., 2016; Morawska & Milton, 2020; Rowe et al., 2021). Therefore, in future pandemic prevention measures, more attention should be paid to indoor environments. For example, the configurations in some indoor environments could be altered to encourage larger social distances, or more conspicuous reminders could be placed to encourage larger IPDs in an indoor room.

The approach pattern showed a significant effect on IPD during the COVID‐19 pandemic. Compared with the active approach, a greater IPD was measured when the participants were passively approached by the confederate, which is consistent with some pre‐pandemic studies (Iachini et al., 2014; Ruggiero et al., 2017) but different from one study conducted by Hsieh and Lee (2021) during the pandemic. In a study by Lee and Chen (2021), IPD was collected via an online survey, and there was no significant difference between the active and passive patterns. The discrepancy may be attributed to the measurement method, in which participants can only judge the distance by the relative position of the characters in a survey instead of experiencing discomfort in a real interaction. However, participants in our experiment could immerse themselves in the virtual environment and naturally interact with the virtual confederate; thus, the real response in the social activities could be collected. The participants showed a larger IPD in the passive pattern because they were asked not to move, which generated feelings of uncertainty and insecurity.

Welsch et al. (2021) conducted an online survey to collect Germans' IPDs before, during, and after the first wave of the pandemic in Germany. The results showed that the distance during the pandemic was 1.5 times to twice the pre‐pandemic distance. A permanently increased IPD was found after the two pandemic waves, showing an asymmetric characteristic of the adaptation to IPD norms. However, their study did not consider the effect of vaccination and other pandemic‐prevention measures. In the current study, the IPDs before COVID‐19 and during the normalization of the pandemic situation were compared in China. An interesting finding was that when people faced the virtual confederate who had received a booster vaccine and wore a face mask, the IPD was the same as before the pandemic in both the active and passive patterns. However, when the participants faced the virtual confederate in other conditions (unmasked or unvaccinated), the IPD was significantly larger than that collected before the pandemic. People's regulation of IPD norms reflects a kind of partial symmetry, which is not entirely consistent with the conclusion of Welsch et al. (2021). One reason for this inconsistency may be that this study considered the factors of anti‐pandemic measures. The recruited participants were all vaccinated with two doses of vaccine. When interacting with confederates who had received a booster vaccine and wore masks, they would perceive their risk of contracting the virus as low and their sense of security as high, leading to the restoration of IPD to the level before the pandemic. Another possibility is that the study was conducted in China, where the pandemic was in its normalization phase, with few daily infection cases at this time. With protection measures in place, the participants felt that interpersonal communication had returned to the state before the pandemic. People's preferences for IPD showed high plasticity and flexibility according to the external environment and situational demands. The influence of COVID‐19 on IPD will gradually decrease until the IPD returns to the normal level seen before the pandemic.

## RESEARCH LIMITATIONS AND FUTURE WORK

This study investigated the effects of anti‐pandemic measures on IPD during the normalization stage of the COVID‐19 pandemic in China, and environmental factors and approach patterns were considered. However, IPD is also influenced by other factors, such as sex, age, culture, etc. The virtual confederate in our experiment was set to male, and sex effects were not evaluated. In the future, the effects of confederates' sex on the IPD in the normalization stage of the COVID‐19 pandemic could be further explored for a more in‐depth study. The recruited participants in this study were young Chinese adults aged 19 to 27 years old. Participants in more age groups and of other cultural backgrounds could be studied to improve the generalizability.

Another limitation of our study is the difference in dimension between the indoor and outdoor conditions. While our findings highlight the influence of environment on IPD, it is important to acknowledge that the outdoor condition was significantly larger than the indoor condition. This difference in dimension may have influenced participants' perception and behaviour, potentially confounding the effects of being outdoors versus indoors. To address this limitation, future studies could aim to create outdoor conditions that are comparable in perceived dimension to the indoor space. This could involve selecting outdoor locations that provide a confined or restricted space similar to the indoor room, or using larger indoor spaces to match the perceived dimension of the outdoor environment. By controlling for the size of the spaces, researchers can better isolate and examine the specific influence of being outdoors or indoors on IPD.

The current study was conducted in China in October 2021, when China was in the stage of pandemic normalization. At that stage, China had implemented the pandemic‐prevention measures of “external defence input and internal defence rebound,” and the daily new infection cases were basically under control (fewer than 0.05 cases per million people). Since December 2022, China has ended its pandemic prevention and control policy, and the pandemic has entered a new stage. Therefore, we recommend that future studies continue to explore IPD under the new pandemic phase in China, so that we can gain a deeper understanding of the effects of the COVID‐19 pandemic on IPD and thus gain more insight into the impact of the pandemic on peoples' lives and social behaviours.

## CONCLUSIONS

This study investigated the effect of wearing masks, receiving vaccinations, approach patterns, and environment on IPD in the normalization stage of the pandemic in China. The IPDs before and during the COVID‐19 pandemic were also compared. The results showed that people chose a smaller IPD when they faced a confederate with a face mask. When they faced a virtual confederate who had received a booster shot, the participants chose the smallest IPD, while the largest distance was found when the participants faced the unvaccinated confederate. Moreover, during the COVID‐19 pandemic, the IPD was larger in the outdoor environment than in indoor rooms. It was interesting to note that when the participants faced a virtual confederate who wore a mask and had received a booster vaccine during the pandemic, the IPD was the same as that collected before the COVID‐19 pandemic in both the active and passive patterns. These findings suggest that IPD is highly adaptive to the pandemic's changing situation. The context of the pandemic and increased exposure to anti‐pandemic measures may impact people's attitudes towards specific objects and further regulate their social behaviors. This study may provide a deeper understanding of the nature of IPD in the pandemic situation and lay the groundwork for future research into human behaviors and social activities during this COVID‐19 crisis.

## CONFLICT OF INTEREST STATEMENT

The authors declare that they have no conflict of interest.

## ETHICS STATEMENT

The experiment contents were approved by the Institutional Review Board of Nanyang Technological University (IRB‐2022‐453).
